# The efficacy of probiotic preparations on inflammatory cytokines in patients with chronic kidney disease: A protocol for systematic review and meta-analysis

**DOI:** 10.1097/MD.0000000000026422

**Published:** 2021-07-02

**Authors:** Peidong Wang, Yanyan Peng, Yueqin Guo, Yongqiang Zhao

**Affiliations:** a Department of General Practice, Gansu Provincial Hospital, Lanzhou, Gansu, China; b Department of Medical, Gansu Provincial Hospital, Lanzhou, Gansu, China; c Department of Urology, Gansu Provincial Hospital of Traditional Chinese Medicine, Lanzhou, Gansu, China.

**Keywords:** chronic kidney disease, inflammatory cytokine, meta-analysis, probiotic preparation

## Abstract

**Background:**

Probiotics supplementation has emerged as adjuvant therapy for chronic kidney disease (CKD) in recent years. However, the effects of probiotic preparations on serum inflammatory cytokine levels are still highly controversial and poorly documented. Therefore, we performed the protocol for systematic review and meta-analysis to further clarify the effects of probiotic preparations in CKD patients.

**Methods:**

This review will develop following the Preferred Reporting Items for Systematic Reviews and Meta-Analyses statement guidelines. We searched literature published until May, 2021 thoroughly in PUBMED, Scopus, EMBASE, Web of Science, and Cochrane Library databases on May, 2021. The risk of bias of included studies was estimated by taking into consideration the characteristics including random sequence generation, allocation concealment, blinding of patients, blinding of outcome assessment, completeness of outcome data, selective reporting, and other bias by Cochrane Collaboration's tool for assessing the risk of bias. Data synthesis and analyses were performed using Stata version 10.0 software.

**Results:**

The results of this systematic review and meta-analysis will be published in a peer-reviewed journal.

**Conclusion:**

We hypothesized that probiotic preparations may decrease the serum levels of inflammatory cytokines and protect the intestinal epithelial barrier of patients with CKD.

## 1 Introduction

Chronic kidney disease (CKD) is a syndrome defined as persistent alterations in kidney structure, function, or both with implications for the health of the individual.^[1,2]^ It is a global health burden with a high economic cost to health systems and affects between 8% and 16% of the population worldwide.^[3]^ Globally, CKD is most commonly attributed to diabetes and/or hypertension, but other causes such as glomerulonephritis, infection, and environmental exposures (such as air pollution, herbal remedies, and pesticides) are common in many developing countries.^[4–6]^ CKD is often underrecognized by patients and clinicians. Early detection and treatment by primary care clinicians are important because progressive CKD is associated with adverse clinical outcomes, including end-stage kidney disease, cardiovascular disease, and increased mortality.

Most CKD patients suffer from the alteration of intestinal microbial flora and the disruption of the intestinal mucosal barrier, which increases the levels of inflammatory cytokines. Cytokines such as interleukin-6 (IL-6) and tumor necrosis factor-α (TNF-α) then mediate systemic inflammatory response by stimulating the production of acute-phase proteins such as C-reactive protein (CRP) and fibrinogen.^[7,8]^ In turn, systemic inflammation accelerates the progression of CKD. Therefore, the intervention on intestinal flora could be a promising treatment for patients with CKD. Probiotics can increase the number of beneficial bacteria in the gut. Published studies have reported that probiotics could delay CKD progression by regulating chronic inflammation and improving renal function.^[9,10]^

Probiotics supplementation has emerged as adjuvant therapy for CKD in recent years because probiotics cost lower and are more acceptable by patients. However, the effects of probiotic preparations on serum inflammatory cytokine levels are still highly controversial and poorly documented. Therefore, we performed the protocol for systematic review and meta-analysis to further clarify the effects of probiotic preparations in CKD patients.

## 2 Methods

### 2.1 Search strategy

This review will develop following the Preferred Reporting Items for Systematic Reviews and Meta-Analyses statement guidelines.^[11]^ We searched literature published until May, 2021 thoroughly in PUBMED, Scopus, EMBASE, Web of Science, and Cochrane Library databases on May, 2021. The search terms included “renal insufficiency,” “chronic kidney diseases,” “chronic renal diseases,” “synbiotics,” “probiotics,” “prebiotics,” “bifidobacterium,” and “lactobacillus.” These terms were searched in the combination of Medical Subject Headings and text words in the title and abstract. Two searchers will independently draft and carry out the search strategy, and the third member will further complete it. The reference list of all selected articles was independently screened by 2 reviewers to identify additional studies left out in the initial search.

The systematic review protocol has been registered on Open Science Framework registries. The registration number is 10.17605/OSF.IO/ZDQ68. Since this study is on the basis of published or registered studies, ethical approval and informed consent of patients are not required.

### 2.2 Eligibility criteria

Two independent researchers removed duplicated articles using EndNote and then screened the titles and abstracts of articles to exclude irrelevant studies. Then they reviewed the full-texts of the remaining records independently to determine eligibility for this meta-analysis according to the following inclusion criteria: (1) randomized controlled trials (RCTs) included crossover, cluster, and quasi-RCT designs; (2) the objects were definitely diagnosed with CKD, and their ages were over 18 years old, regardless of hemodialysis; (3) the intervention group was supplemented with probiotic or probiotic derivatives in any form, while the control group was given placebo or conventional treatment; and (4) the studies included 1 of the following indicators: urea, creatinine, uric acid, paracresyl sulfate (PCS), indoxyl sulfate, IL-6, CRP, and TNF-α. Any inconsistencies were resolved by consulting the third researcher.

### 2.3 Exclusion criteria

Any one of the following articles can be excluded: (1) observational studies, animal experiments, case reports, reviews, and other non-RCTs; (2) the full text cannot be obtained; (3) a lack of complete data; and (4) patients who had been diagnosed with significant digestive tract disease, received kidney transplantation, and used antibiotics recently.

### 2.4 Data extraction

Two reviewers will be responsible for information extraction according to the following information: the basic information of the included studies, including the first author, the year of publication, etc. The basic characteristics of the subjects, including the number of patients in the treatment group and the control group, gender composition, average age, intervention drug dosage, treatment course, and other specific details. The primary measured outcome was urea toxins. The secondary outcomes were creatinine, uric acid, PCS, indoxyl sulfate, IL-6, CRP, and TNF-α.

### 2.5 Assessment of risk of bias

The risk of bias of included studies was estimated by taking into consideration the characteristics including random sequence generation, allocation concealment, blinding of patients, blinding of outcome assessment, completeness of outcome data, selective reporting, and other bias by Cochrane Collaboration's tool for assessing the risk of bias.^[12]^ Quality of evidence was appraised with the Grading of Recommendation Assessment, Development, and Evaluations (GRADE) method including the risk of bias, inconsistency, indirectness, imprecision, and publication bias by the GRADEpro GDT 2015 to create SoF table. Quality assessment and GRADE were independently performed by 2 researchers. Disagreements over the risk of bias in particular studies will be resolved by discussion, which routinely implicated the third researcher if necessary.

### 2.6 Statistical analysis

We performed the meta-analysis by Stata version 10.0 software and calculated the statistics using the inverse variance statistical method. Continuous variables were expressed as the weighted mean difference or standardized mean difference and 95% confidence interval. Weighted mean difference was used when data were measured on the same scale and standardized mean difference was used if data were measured using different scales. Heterogeneity among the studies was quantified with the *I*^2^ statistic. If *I*^2^ > 50% or *P* < .1, a random effect model was used to decrease heterogeneity, and the subgroup and sensitivity analysis were performed to explore the sources of heterogeneity. Otherwise, heterogeneity was negligible and a fixed-effect model was used. Publication bias was assessed using Egger linear regression test, and *P* < .05 indicated no publication bias among the included studies.

## 3 Discussion

The theory of the gut-kidney axis, raised by Khoury et al,^[13]^ indicates the interaction between the gastrointestinal tract and renal. This theory also suggests the changes in the intestinal microbiome and intestinal epithelial barrier in CKD patients, which result in intestinal microbial dysbiosis. It is mainly manifested as a decrease in probiotics and the expansion of endotoxin-producing bacteria and metabolic toxin-producing bacteria. Recent studies revealed the importance of the gut microbiota in the development and progression of CKD.^[14,15]^ Dysbiosis of the intestinal microbiota increases urea toxins, such as indole-3 acetic acid, PCS, and indoxyl sulfate, which damage the epithelial tight junctions and increase the permeability of the intestinal wall via endotoxemia and systemic inflammation.^[16]^ As a consequence, intestinal endotoxins may go through the intestinal wall into the blood circulation, induce microinflammation in the kidney and cause renal endothelial dysfunction, fibrosis, and tubular damages, which subsequently accelerates the decline of renal function. This current meta-analysis summarizes the effects of probiotic preparations on serum levels of uremic toxin and specific inflammatory cytokines in CKD patients comprehensively. Long-term clinical trials with a larger sample size are still needed to verify the present study results further.

## Author contributions

Yongqiang Zhao: study design. Peidong Wang: data collection and original draft. Yueqin Guo: review draft; Yanyan Peng: edit language. All of the authors approved the submission.

**Conceptualization:** Yanyan Peng.

**Data curation:** Yanyan Peng.

**Funding acquisition:** Yongqiang Zhao.

**Investigation:** Yueqin Guo.

**Methodology:** Yueqin Guo.

**Writing – original draft:** Peidong Wang.
